# Denatonium inhibits RANKL-induced osteoclast differentiation and rescues the osteoporotic phenotype by blocking p65 signaling pathway

**DOI:** 10.1186/s10020-024-01031-2

**Published:** 2024-12-19

**Authors:** Sheunghun Lee, Hyerim Lee, You-Jee Jang, Kyubin Lee, Hye-Jung Kim, Jung Yeol Lee, Jin-Man Kim, Sunyou Park, Jin Sook Song, Ji Hoon Lee, Tae Kyung Hyun, Jae-Il Park, Sun-Ju Yi, Kyunghwan Kim

**Affiliations:** 1 Department of Biological Sciences and Biotechnology, Chungbuk National University, Cheongju, Chungbuk Republic of Korea; 2 Department of Biomedical Laboratory Science, Honam University, Gwangju, Republic of Korea; 3 New Drug Development Center, KBIO Osong Medical Innovation Foundation, Chungbuk, Republic of Korea; 4 New Drug Development Center, Daegu-Gyeongbuk Medical Innovation Foundation, Daegu, Republic of Korea; 5 Asan Medical Center, Asan Institute for Life Sciences, Seoul, Republic of Korea; 6 Data Convergence Drug Research Center, Therapeutics & Biotechnology Division, Korea Research Institute of Chemical Technology, Daejeon, Republic of Korea; 7 Department of Industrial Plant Science & Technology, Chungbuk National University, Cheongju, Chungbuk Republic of Korea; 8 Korea Basic Science Institute, Gwangju Center at Chonnam National University, Gwangju, Republic of Korea

**Keywords:** Denatonium, Osteocastogenesis, p65, Osteoporosis

## Abstract

**Background:**

Bone remodeling is a critical process that maintains skeletal integrity, orchestrated by the balanced activities of osteoclasts, which resorb bone, and osteoblasts, which form bone. Osteoclastogenesis, the formation of osteoclasts, is primarily driven by NFATc1, a process activated through c-Fos and NF-κB signaling pathways in response to receptor activator of nuclear factor κB ligand (RANKL). Dysregulation of RANKL signaling is a key contributor to pathological bone loss, as seen in conditions such as osteoporosis.

**Methods:**

We investigated the effects of denatonium, a known bitter compound, on RANKL-induced osteoclast differentiation. We used RNA sequencing (RNA-seq) to analyze gene expression profiles in osteoclast precursors treated with denatonium. Transcription factor prediction analysis was conducted to identify key targets of denatonium action. Additionally, we performed Western blotting to examine the phosphorylation status of AKT and p65, crucial components of the NF-κB pathway. Chromatin immunoprecipitation (ChIP) assays were employed to assess the binding of p65 to promoter regions of osteoclast-related genes. Finally, we tested the therapeutic potential of denatonium in a mouse model of osteoporosis.

**Results:**

Our findings demonstrated that denatonium significantly inhibited RANKL-induced osteoclastogenesis by targeting the p65 pathway. RNA-seq analysis revealed a downregulation of osteoclast-related genes following denatonium treatment, corroborated by transcription factor prediction analysis, which highlighted p65 as a key target. Denatonium effectively blocked the phosphorylation of AKT and p65, key steps in NF-κB activation. ChIP assays further confirmed that denatonium reduced the enrichment of p65 at promoter regions critical for osteoclast differentiation. In vivo, denatonium treatment in an osteoporosis animal model led to a significant restoration of bone health, demonstrating its potential as a therapeutic agent.

**Conclusions:**

This study identifies denatonium as an inhibitor of RANKL-induced osteoclast differentiation, potentially acting through suppression of the p65 signaling pathway. The ability of denatonium to downregulate osteoclast-related genes and inhibit key signaling events highlights its potential as a candidate for further investigation in the context of bone loss and osteoporosis.

**Supplementary Information:**

The online version contains supplementary material available at 10.1186/s10020-024-01031-2.

## Background

Throughout our lives, bone tissue is maintained in a delicate balance between bone resorption and bone formation. After the osteoclasts remove old or damaged bone, osteoblasts take over and form new bone tissue. Osteoporosis develops when bone resorption surpasses bone formation. This imbalance ultimately decreases bone mineral density and bone mass, leading to a decrease of bone strength and an increased risk of bone fractures (Sozen et al. 2017; Compston et al. 2019).

Osteoclastogenesis is a complex process that involves the specific regulation by intracellular signaling pathways. Macrophage colony-stimulating factor (M-CSF) and receptor activator of nuclear factor-kappa B ligand (RANKL) are involved in the survival and differentiation of osteoclast precursor (OCP) cells. RANKL binds to its receptor, RANK, on OCP cells, and activates various downstream signaling pathways, including the NF-κB pathway. This results in the expression of osteoclast-specific genes, such as nuclear factor of activated T cells c1 (*Nfatc1*), matrix metalloproteinase 9 (*Mmp9*), tartrate-resistant acid phosphatase (*Trap*), dendrocyte expressed seven transmembrane protein (*Dcstamp*), and *cathepsin K* (Maeda et al. 2022; Udagawa et al. 2021). Recently, we reported the anti-osteoporotic activity of an ethyl acetate fraction (LEA) of shiitake mushroom (*Lentinula edodes*), which suppressed RANKL-induced osteoclastogenesis (Lee et al. 2020a, b); however, the precise molecular mechanisms underlying its preventative effects on osteoporosis are unknown.

In this study, we identified denatonium, a small cation, in the ethyl acetate fraction of shiitake mushroom using liquid chromatography coupled with tandem mass spectrometry (LC-MS/MS) analysis. Denatonium effectively inhibited RANKL-induced osteoclast formation. RNA-seq analysis revealed that denatonium reduced expression of osteoclast-related genes, such as *Dc-stamp*, *Mmp9*, and *Nfatc1*. Additionally, denatonium treatment inhibited AKT-NF-ĸB signalling pathway and further impeded the p65 localization at the promoter regions of target genes, as evidenced by chromatin immunoprecipitation (ChIP) analysis, impacting osteoclast differentiation. Moreover, denatonium showed potential in preventing the osteoporotic phenotype induced by ovariectomy or glucocorticoid treatment. Collectively, our findings reveal a previously undocumented role for denatonium in modulating bone homeostasis and highlight its potential as a therapeutic candidate for osteoporosis.

## Methods

### Antibodies

The antibodies used in this study were as follows: antibodies for p65 (western blotting), phosphor p65(ser536), p50/p150, p52/p105, and RELB were from Cell Signaling Technology (Danvers, MA, USA); antibodies for lamin A/C and β-actin were from Santa Cruz (Santa Cruz, CA, USA); antibodies for phospho AKT, AKT, p38, phospho ERK, ERK, and IκBα were from Cell Signaling Technology (Danvers, MA, USA); antibody for phospho p38 was from EMD Millipore (Temecula, CA, USA); p65 antibody (ChIP) was from Diagenode (Denvile, NJ, USA).

### Identification and quantification of denatonium

The samples underwent filtration using a 0.45 μm pore size membrane filter, and subsequent analysis was carried out using an Accelar UHPLC system (Thermo Fisher Scientific, USA). Chromatographic separation was achieved employing an ACQUITY UPLC column (BEH C18: 2.1 × 50 mm, 1.7 μm) with a flow rate of 0.4 ml/min. A gradient elution method was employed, utilizing two mobile phases: mobile phase A containing 0.1% (v/v) formic acid, and mobile phase B consisting of acetonitrile with 0.1% (v/v) formic acid. Mass spectrometric analysis was performed using a LTQ-Orbitrap XL mass spectrometer system (Thermo Fisher Scientific, USA). ion mode. Data analysis was conducted using the Xcalibur software (Thermo Fisher Scientific, USA), along with the ChemSpider database (http://www.chemspider.com/).

### Mice

All C57BL/6J mice were housed in a controlled facility under a 12-hour light/dark cycle at 22 °C. All procedures were approved by the Institutional Animal Management and Use Committee of Chungbuk National University (CBNUA-1245-19-02). Age-matched male mice were used for the isolation of osteoclast precursors, while age-matched female mice were utilized for ovariectomy experiments.

### Ovariectomy and micro-CT analysis

For the ovariectomy (OVX) experiments, 2-month-old female C57BL/6 mice (approximately 20 g) were randomly assigned to four groups (*n* = 5): sham-operated mice, bilateral OVX mice treated with vehicle, and OVX mice treated with denatonium at doses of 1.5 mg/kg and 4.5 mg/kg. Following the procedure, mice received intraperitoneal injections of either vehicle or denatonium once a week for 8 weeks. For micro-CT analysis, trabecular bone parameters were analyzed by Analyze 12.0 software (AnalyzeDirect, KS, USA), and the distal femur was scanned using a Quantum GX micro-CT imaging system (PerkinElmer, MA, USA), and (Lee et al. 2020a, b).

### Osteoclast precursor (OCP) cells isolation, TRAP staining, and bone resorption assay

OCP cells were prepared according to a previously described method (An et al. 2014). Bone marrow cells were obtained from the femurs and tibias of 7-week-old C57BL/6J mice and grown in minimum essential medium-α (MEM-α) with 10% FBS and 5 ng/ml M-CSF for 16 h. The nonadherent cells were then cultured with 30 ng/ml M-CSF for an additional 3 days. After removing the floating cells, the adherent cells were designated as OCP cells.

For osteoclast differentiation, OCP cells were seeded into 48-well plates and treated with 30 ng/mL of M-CSF (IKIM Bio, South Korea) and 100 ng/mL of RANKL (IKIM Bio, South Korea), either alone or in combination with denatonium. After 3 to 4 days, the cells were fixed with 3.7% formaldehyde and stained for tartrate-resistant acid phosphatase (TRAP) using an acid phosphatase leukocyte kit (Sigma-Aldrich, MO, USA). TRAP-positive, multinucleated cells with three or more nuclei were identified as osteoclasts and counted under a light microscope (Kim et al. 2016). To assess the bone resorption activity of the osteoclasts, OCP cells were differentiated on dentin slices (IDS, United Kingdom) in the presence or absence of denatonium at concentrations of 100 µM and 200 µM for 7 to 10 days. Afterward, the osteoclasts were removed from the bone slices by sonication, and the resorption pits formed on the slices were stained with Mayer’s hematoxylin (Sigma-Aldrich, MO, USA). The areas of these pits were quantitatively analyzed using ImageJ software.

### Zebrafish maintenance and whole-mount skeletal staining

Zebrafish (wild-type strain) were kept in a circulating water system at 28℃, following a day-night cycle of 14 h light and 10 h dark. They were fed live brine shrimp twice daily. All experimental procedures were approved by the Animal Care and Use Committee of Chungbuk National University (CBNUA-1391-20-01). Following fertilization, embryos were maintained at 28 °C under a 14-hour light/10-hour dark cycle. At 10 days post-fertilization (dpf), larvae were exposed to the specified concentration of denatonium alongside 25 µM prednisolone. Whole-mount bone staining of zebrafish larvae was conducted using Alizarin red, as previously described (Aceto et al. 2015).

### RNA sequencing

Total RNA was extracted using Tri-reagent (Favorgen, Tiwan). Libraries were prepared from 2 µg of total RNA using the SMARTer Stranded RNA-Seq Kit (Clontech Laboratories, Inc., CA, USA). We performed high-throughput sequencing on a HiSeq 2500 instrument with a paired-end 100 sequencing approach (Illumina, Inc., CA, USA). Analysis of mRNA-seq was performed as previously described (Lee et al. 2020a, b; Yi et al. 2021, 2023).

### Real-time quantitative PCR

Total RNA was converted to cDNA using the Molony Murine Leukemia Virus(M-MLV) reverse transcriptase (Promega, WI, USA). Real-time PCR was performed using IQ SYBR Green Supermix in an IQ5 real-time thermal cycler (Bio-Rad, CA, USA). *β-actin* mRNA levels were used to normalize the expression levels of the target gene mRNAs among samples. The primer sequences utilized in qPCR are shown in Table S1.

### Measurement of intracellular reactive oxygen species (ROS)

Cells were cultured with or without RANKL (100 ng/ml) in the presence or absence of denatonium (200 µM) on 6-well plates for 2 days. Cells were starved in phenol red-free MEM-α for 1 h and treated with RANKL (100 ng/ml) for 5–10 min. CellROX™ Deep Red Reagent (Thermo Fisher Scientific, MA, USA) was added to a final concentration of 0.5 µM for 30 min before harvesting and detection. Signals were measured at 660 nm by flow cytometry (CytoFlex, Beckman Coulter, USA), and the results are presented as the mean fluorescence intensity (MFI) of three independent experiments(Yi et al. 2023).

### Measurement of ATP levels

ATP levels were quantified using a luciferin-luciferase based assay with the ENLITEN ATP Assay System Bioluminescence Detection Kit (Promega, USA), following the manufacturer’s instructions. Briefly, OCP cells were seeded in 12-well plates with M-CSF (30 ng/ml) and RANKL (100 ng/ml) in MEM-α complete media, with or without denatonium (200 µM). After 1 day, cells were washed with PBS and lysed with the lysis buffer. Unnecessary components were precipitated using 2% trichloroacetic acid, and supernatants were neutralized by adding Tris-Acetate buffer. A 50 µl aliquot of luciferase reagent was then added to 50 µl of the neutralized sample, and ATP levels were measured immediately using a luminometer.

### Reporter gene assay

Reporter gene assays were performed as previously described (Lee et al. 2020a, b). In brief, NFATc1-Luc plasmids were transfected with vectors for p65, c-FOS, or NFATc1 into 293T cells for 24 h. Cells were lysed in Reporter Lysis buffer (Promega, USA) and measured luminescence using SpectraMax i3x (Molecular Devices, CA, USA).

### Chromatin immunoprecipitation (ChIP) assay

Cells were cross-linked using 1% formaldehyde for 10 min. Cell lysates were prepared with a hypotonic buffer (10 mM HEPES-KOH, pH 7.8, 10 mM KCl, 1.5 mM MgCl2, and protease inhibitors) and subsequently sonicated using a Bioruptor (Diagenode, USA) for 20 cycles. After preclearing, ChIP assays were conducted using antibodies targeting p65 and control IgG (An et al. 2014; Yi et al. 2021; Kim et al. 2018). The DNA obtained from the precipitation step was subjected to real-time quantitative PCR with primers designed for specific regions of the *Mmp9* promoter, *Nfatc1* promoter, and *Dcstamp* promoter. Sequences of the primers used for quantitative real time PCR are shown in Table S1.

### Statistical analysis

Statistical analyses were performed in GraphPad Prism 10 (GraphPad Software Inc., CA, USA). Quantitative data were expressed as either mean ± SD or the mean ± SEM values, with the specific number of replicates (n) are indicated in the figure legends. The significance of differences was evaluated using two-tailed t-test, one-way ANOVA, or two-way ANOVA, followed by post-hoc comparisons using Dunnett’s or Tukey’s multiple comparison tests, depending on the number of groups being compared. ns, not significant; *P < 0.05; **P < 0.01; ***P < 0.001; ****P < 0.0001. All experiments were independently repeated at least two to three times, and consistent results were obtained.

## Results

### Denatonium inhibits RANKL-mediated osteoclastogenesis

**Fig. 1:**
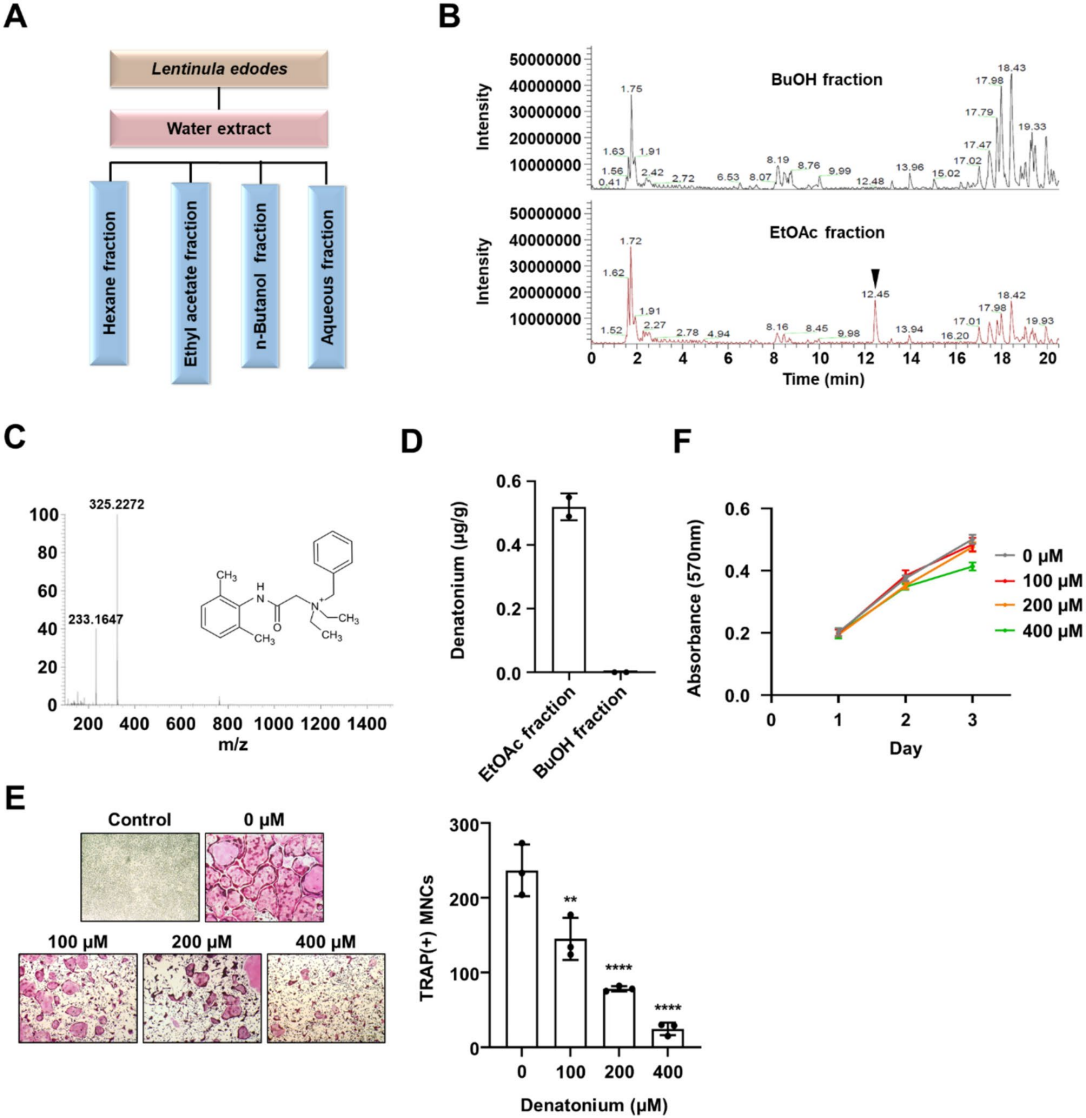
Denatonium, a bioactive molecule isolated from *Lentinula erodes*, inhibits osteoclast differentiation. **A** A schematic representation outlining the procedure for extracting and fractionating antiosteoclastic compounds from *L. edodes*. **B** LC-MS chromatograms of the butanol and ethyl acetate fractions. **C** Identification of denatonium from an EtOAc fraction using LC-MS/MS. **D** Comparison of denatonium content in two fractions (BuOH and EtOAc). **E** Representative images of TRAP-stained cells treated with various concentrations of denatonium. One-way ANOVA followed by Dunnett’s multiple comparisons test, Mean ± SD of three independent experiments. **P < 0.01; ****P < 0.0001. **F** Effect of denatonium on OCPs proliferation

Our previous studies (Lee et al. 2020a, b) revealed the anti-osteoporotic activity of LEA through inhibition of osteoclast differentiation. To further explore potent anti-osteoporotic compounds derived from *L. edodes*, LEA was separated and analyzed by LC-MS/MS. By comparing the mass spectra of EtOAc with n-butanol fractions, we identified a distinct compound known as denatonium (Fig. 1A-C). To confirm and quantify denatonium from *L. edodes*, these two fractions were analyzed using high performance liquid chromatography. Denatonium was present in the EtOAc fraction, but not in the n-butanol fraction (Fig. 1D). Next, we demonstrated that denatonium potently inhibited osteoclast differentiation with a half-maximal effective concentration (EC50) of 200 µM (Fig. 1E). In contrast, denatonium did not significantly affect OCP cell proliferation (Fig. 1F), suggesting that denatonium selectively inhibits osteoclast differentiation. We also determined the effect of denatonium on osteoblast differentiation and found that it slightly increased alkaline phosphatase (ALP) activity and calcium deposition (Figure S1A and B). Additionally, denatonium treatment modestly upregulated the expression of osteoblast differentiation-related genes, including *Alp*, *Col1a1*, and *Runx2* (Figure S1C). These findings suggest that denatonium not only inhibits osteoclast differentiation but also modestly promotes osteoblast differentiation, potentially contributing to enhanced bone formation.

### Denatonium modulates the expression of osteoclast-related genes

**Fig. 2:**
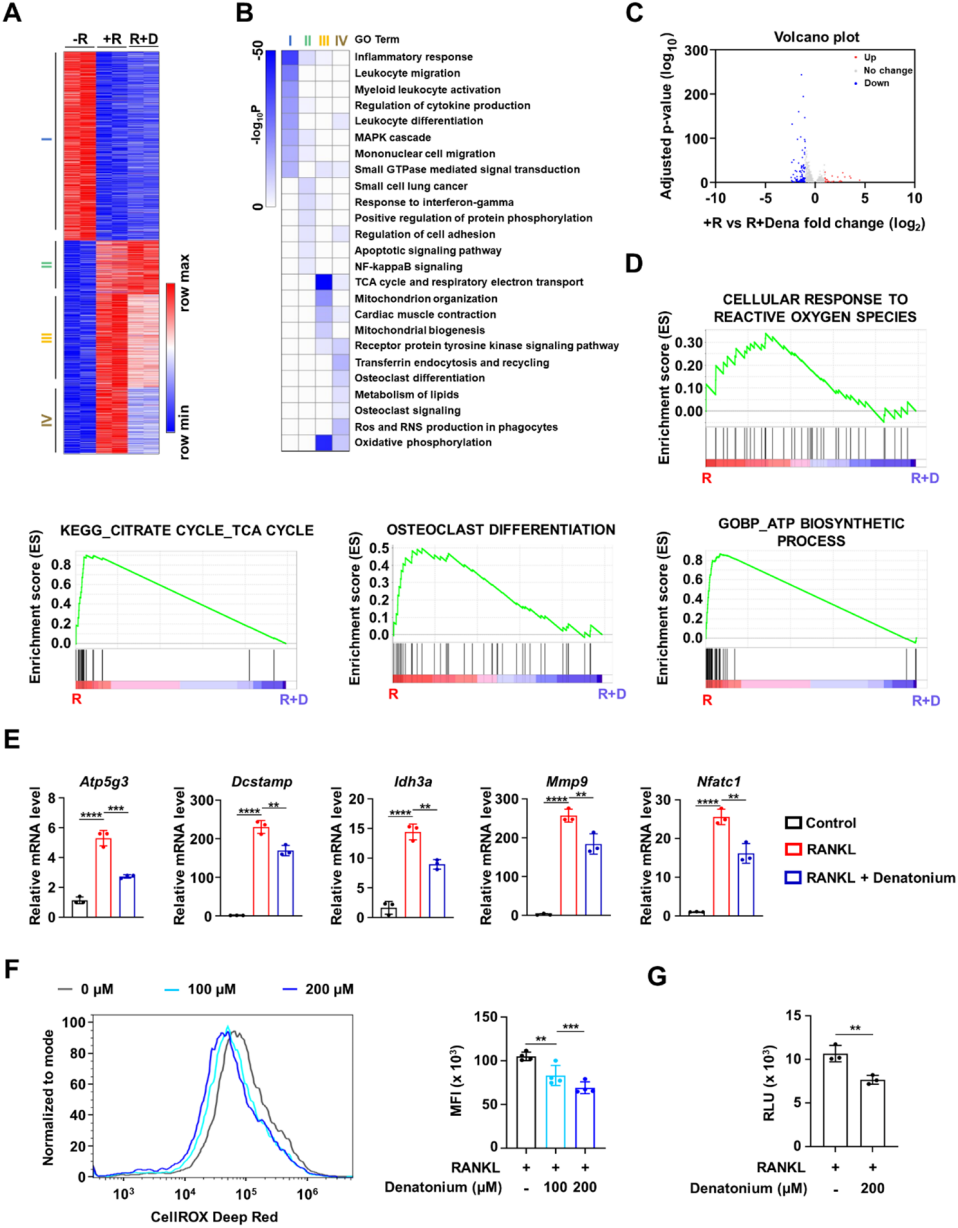
Denatonium alters gene expression profiling in osteoclast precursor (OCP) cells. **A** K-means (K = 4) clustering of 2,687 DEGs for any pairwise comparison among three conditions [− R; no RANKL, +R; RANKL (100ng/ml), R + D; RANKL with denatonium (400µM)]. OCP cells were exposed to RANKL (100ng/ml) for 72 h, with or without the presence of denatonium. **B** Heatmap showing the p-value significance of GO term enrichment for genes in each cluster. **C** Volcano plot of transcriptomic changes between + R and R + D. Genes with increased (red) or decreased (blue) expression in R + D-treated cells relative to RANKL-treated cells defined based on an FDR-adjusted *p* < 0.05 and > 2-fold expression change. **D** GSEA of 2,687 genes as in **A** shows the enrichment of genes associated with osteoclast differentiation, ATP biosynthetic process, TCA cycle, and cellular response to ROS. **E** qRT-PCR was performed to quantitate relative mRNA levels of representative genes for **D**. One-way ANOVA followed by Tukey’s multiple comparisons test, Mean ± SD of three independent experiments. *P < 0.05; **P < 0.01; ***P < 0.001; ****P < 0.0001; ns, not significant. **F** Flow cytometry histograms were used to measure cellular ROS levels using CellROX deep red. The bar graphs represent the MFI of OCP cells stimulated by RANKL (100 ng/ml, 10 min) with or without denatonium (200 µM). One-way ANOVA followed by Dunnett’s multiple comparisons test, Mean ± SD of three independent experiments. **P < 0.01; ***P < 0.001. **G** OCP cells were incubated with denatonium (200 µM) in the presence of RANKL (100 ng/ml). Cellular ATP levels were measured. Two-tailed *t*-test, Mean ± SEM of three independent experiments. **P < 0.01

We next sought to identify the genes regulated by denatonium treatment during osteoclast differentiation. Total mRNA from control or RANKL-induced osteoclasts treated with denatonium was used for RNA sequencing A comparative transcriptome analysis revealed 2,729 genes that were differentially expressed (Table S2). Based on fold-change and p-values, the differentially expressed genes were classified into four clusters (Fig. 2A). To determine the putative function of each cluster, we performed a gene ontology (GO) analysis. As shown in Fig. 2B, each cluster was associated with a specific GO term: cluster I, inflammation response; cluster II, response to interferon-gamma; cluster III, tricarboxylic acid (TCA) cycle and respiratory electron transport; cluster IV, ROS production and osteoclast differentiation. Given that RANKL stimulates the expression of osteoclast-related genes and denatonium inhibits RANKL-mediated osteoclast formation, we focused on RANKL-induced genes. A total of 142 genes were differentially expressed following denatonium treatment, with 27 upregulated and 115 downregulated. (Fig. 2C). Furthermore, gene set enrichment analysis (GSEA) scoring plots and their corresponding heat maps indicated a significant enrichment in the cellular response to ROS and osteoclast differentiation (Fig. 2D). GO analysis results were further verified by qRT-PCR (Fig. 2E).

RANKL-induced ROS production facilitates osteoclast differentiation(Yi et al. 2023; Ha et al. 2004; Kang and Kim 2016; Lee et al. 2005; Sasaki et al. 2009). Our transcriptome analysis revealed that denatonium regulates the expression of genes associated with ROS production and osteoclastogenesis. Therefore, it is likely that denatonium attenuates RANKL-mediated osteoclast formation by decreasing ROS production. We measured ROS levels in RANKL-treated s with or without denatonium and found that ROS levels were significantly decreased by denatonium treatment (Fig. 2F). Additionally, genes involved in ATP production, a critical factor for osteoclast fusion and differentiation (Kim et al. 2007; Takegahara et al. 2024) were reduced by denatonium. We analyzed ATP concentrations following denatonium treatment and observed that denatonium treatment apparently decreased ATP levels (Fig. 2G).

### Denatonium negatively regulates NF-ĸB transactivity by blocking AKT signaling pathway

**Fig. 3:**
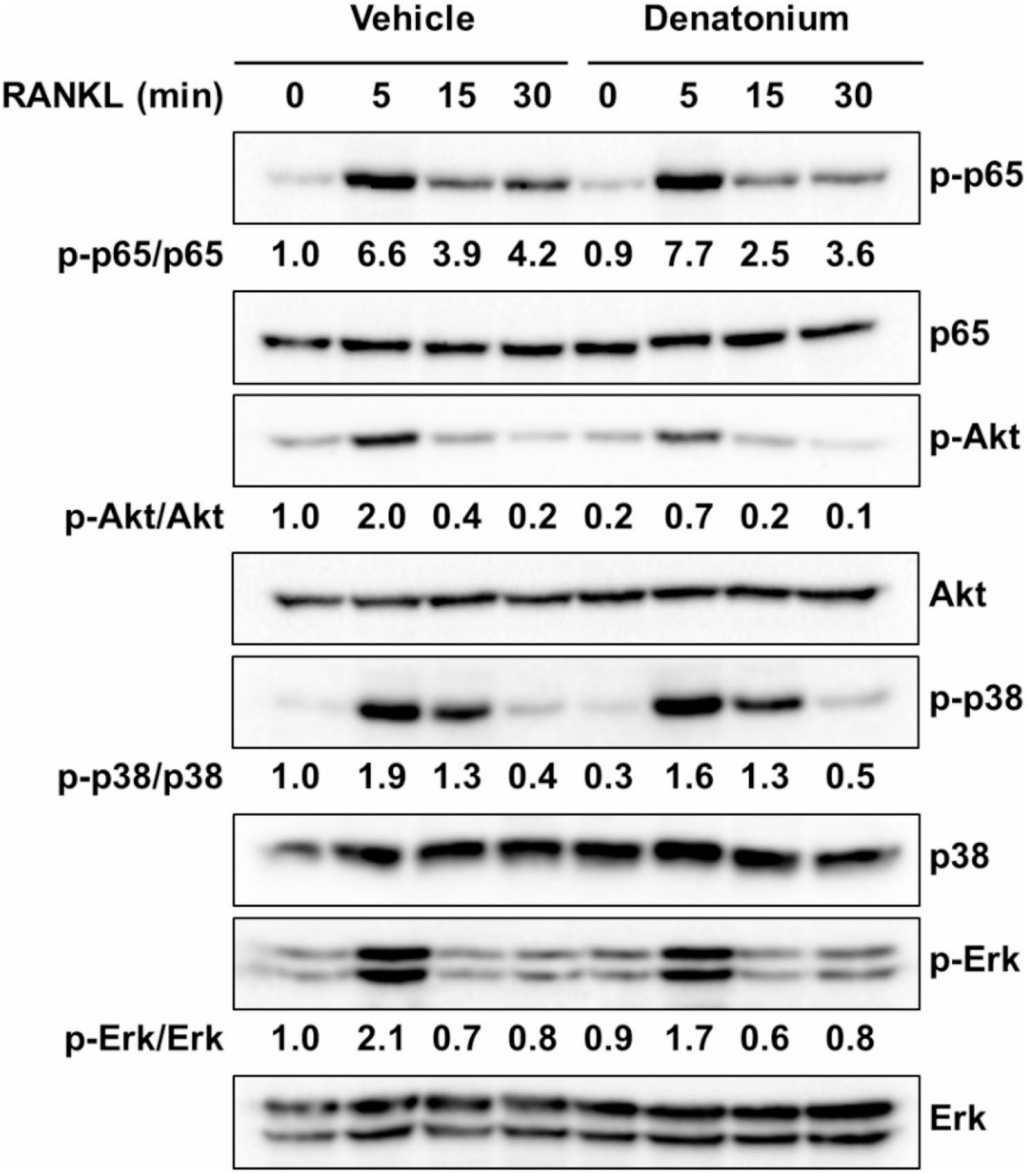
Denatonium inhibits the phosphorylation of AKT and p65. Effect of denatonium on the AKT, MAPK, and NF-κB signaling pathways in response to RANKL treatment. OCP cells were pretreated with denatonium (200 µM) alongside RANKL (100 ng/ml) for the specified durations. Whole cell lysates were subjected to western blot analysis using the appropriate antibodies. The intensity of the western blot bands was quantified using ImageJ software, and results are presented as the relative ratio of each protein band intensity normalized to the total protein band intensity

The binding of RANKL to the RANK receptor activates diverse signaling pathways, including MAPKs, AKT, and NF-κB, which facilitate osteoclast formation (Lee et al. 2016; Asagiri and Takayanagi 2007). To investigate the effect of denatonium on the RANKL signalling pathway, OCPs were pre-treated with denatonium for 1 h and then stimulated with RANKL for the specified times. Phosphorylation of AKT and p65 decreased in denatonium-treated OCP cells (Fig. 3). However, denatonium did not affect the RANKL-induced phosphorylation of p38 and ERK.

**Fig. 4:**
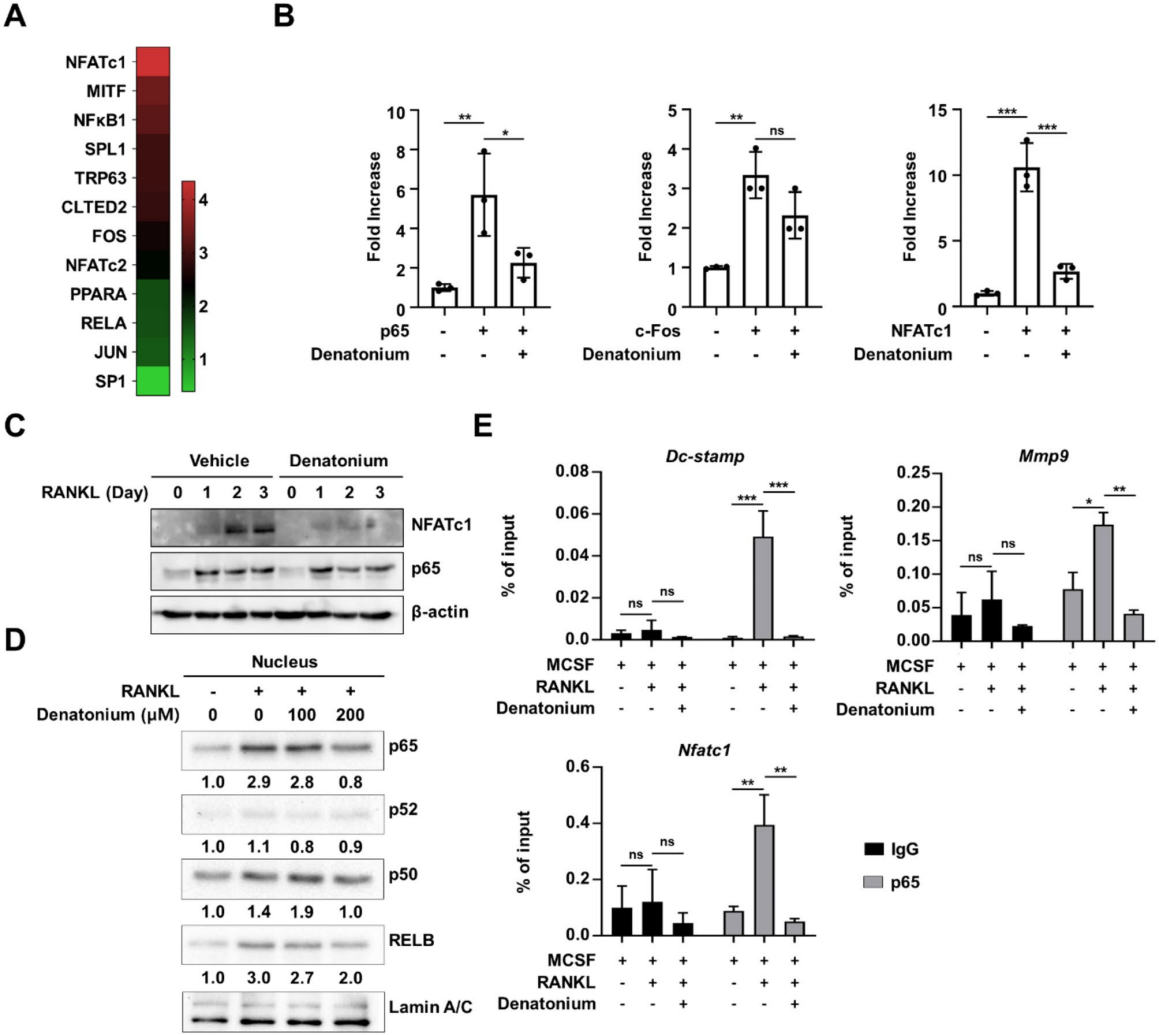
Denatonium blocks the nuclear translocation and recruitment of p65 at osteoclastogenic genes. **A** Analysis of transcription factors governing the expression of genes in cluster 4 as shown in Fig. 2A, using TRRUST v2. **B** 293T cells were transfected with the reporter plasmid Nfatc1-Luc along with p65, c-Fos, or Nfatc1, with or without denatonium (200 µM). Each bar represents the Means ± S.D. of three independent experiments, one-way ANOVA followed by Tukey’s multiple comparisons test. *P < 0.05; **P < 0.01; *** *p* < 0.001; ns = not significant. **C** Effect of denatonium on NFATc1 expression. OCO cells were cultured with or without denatonium (200 µM) in the presence of M-CSF (30 ng/ml) and RANKL (100 ng/ml). Whole-cell lysate were analyzed by immunoblotting with NFATc1 and p65 antibodies. β-Actin served as the loading control. **D** Effect of denatonium on NF-κB nuclear translocation. Nuclear fractions of OCPs were prepared after RANKL (100 ng/ml, 30 min) treatment with vehicle or denatonium and nuclear translocation of NF-κB was measured by western blot analysis with the indicated antibodies. The western blot band was quantified using Image J software and shown as the relative ratio of each protein band intensity normalized to Lamin A/C band intensity. **E** ChIP-qPCR of p65 enrichment at the *Mmp9* (− 0.5 kb), *Nfatc1* (− 0.7 kb), and *Dcstamp* (− 0.6 kb) promoters following RANKL treatment (100 ng/ml, 30 min) with or without denatonium (200 µM). Two-way ANOVA followed by Tukey’s multiple comparisons test, Mean ± SD of three independent experiments. *P < 0.05; **P < 0.01; ***P < 0.001; ns, not significant

Next, we identified transcription factors that control the expression of genes correlated with denatonium during osteoclastogenesis. Using the TRRUST v2 database, we analyzed a set of 445 genes regulated by denatonium. As shown in Fig. 4A, several transcription factors essential for osteoclast differentiation and *Nfatc1* expression, including NFATc1, NF-ĸB, and c-Fos, were highly ranked(Yi et al. 2021; Boyce et al. 2015; Abu-Amer 2013; Novack 2011; Alles et al. 2010). To examine whether denatonium affects the transactivities of c-FOS, p65, and NFATc1 to the *Nfatc1* promoter, we conducted an Nfatc1 luciferase assay (Fig. 4B). Denatonium significantly inhibited the transactivation of p65 and NFATc1, while having a modest inhibitory effect on c-Fos transactivation. We also observed that denatonium completely abrogated the protein expression of NFATc1, but not p65 (Fig. 4C).

Given that NF-κB is an upstream regulator of NFATc1, we evaluated how denatonium influences the p65 signaling pathway. OCP cells were stimulated with RANKL for 30 min in the presence or absence of denatonium and the nuclear localization of NF-κB was determined by western blot analysis. Denatonium treatment resulted in a significant reduction in the RANKL-induced nuclear translocation of p65, whereas the expression of p65 was unchanged (Fig. 4D and Figure S2). Moreover, ChIP experiments using p65 antibody revealed stable occupancy of the promoter regions of target genes in response to RANKL treatment. However, denatonium treatment compromised p65 enrichment in the target genes (Fig. 4E). Based on our present data and the previous findings that AKT is known to activate p65 pathway (Yang et al. 2023; Tang et al. 2022; Bai et al. 2009; Sakurai et al. 2003; Kwon et al. 2016), we suggest that denatonium negatively regulates p65 signaling via the AKT signaling pathway.

### Denatonium prevents bone loss in an osteoporosis animal model

**Fig. 5:**
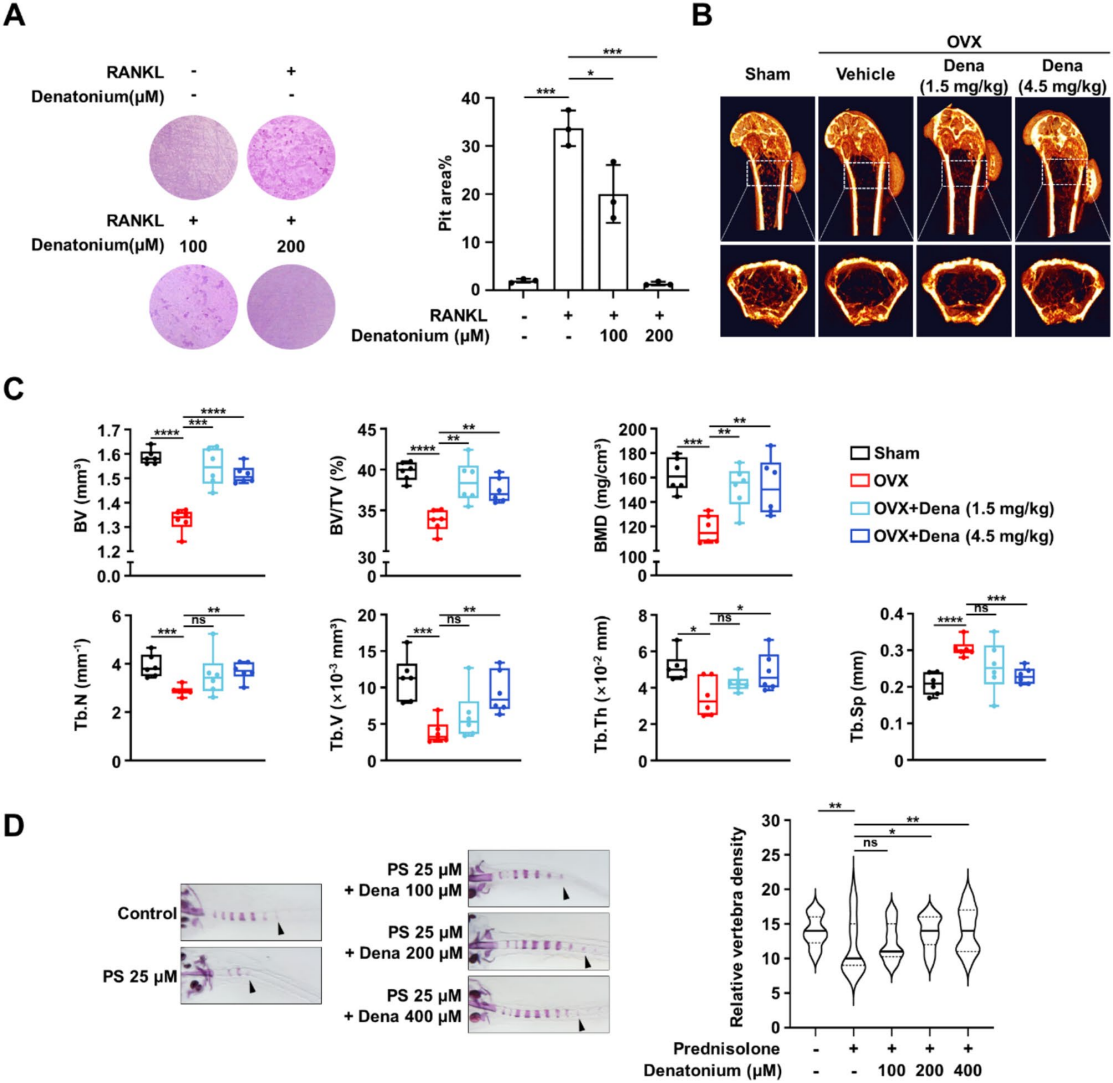
Denatonium prevents bone loss in osteoporotic models. **A** Bone-resorption activity of OCPs treated with vehicle or denatonium (100 µM and 200 µM) with or without RANKL (100 ng/mL) for 10 d. One-way ANOVA analysis, Mean ± SD of three independent experiments. *P < 0.05; ***P < 0.001. **B–C** Micro-CT analysis of the femurs of a two-month-old sham-operated, saline-treated (Sham), saline-treated OVX (OVX), and denatonium-treated OVX (OVX + Dena 1.5 mg/kg or 4.5 mg/kg) mice (*n* = 6). BMD, bone mineral density; BV, bone volume; BV/TV, trabecular bone volume per tissue volume; Tb.Th, trabecular thickness; Tb. Sp, trabecular spacing; Tb.N, trabecular number; Tb.V, trabecular volume. Two-tailed *t*-test, Mean ± SEM. *P < 0.05; **P < 0.01; ***P < 0.001; ****P < 0.0001. **D** The larvae at 10 d post-fertilization were treated with 25 µM prednisolone (PS) and varying denatonium concentrations (0, 100, 200, and 400 µM) for 3 d. Whole-mount Alizarin red staining was performed to analyze the mineralized bone. The relative density of vertebral bones was evaluated by measuring the areas of the initial five stained vertebrae (indicated by arrowhead). One-way ANOVA followed by Tukey’s multiple comparisons test, Mean ± SEM (*n* = 36). *P < 0.05; **P < 0.01; ns, not significant

To determine the effect of denatonium on the bone resorption ability of osteoclasts, we conducted a pit formation assay. As expected, denatonium treatment significantly decreased RANKL-induced pit formation (Fig. 5A). We further evaluated the potential effects of denatonium on osteoporosis in vivo. In ovariectomized mice, the administration of two different doses of denatonium led to an increase in bone mineral density (BMD), trabecular thickness (Tb.Th), trabecular number (Tb.N), and trabecular volume (Tb.V), while reducing trabecular separation (Tb.Sp) when compared to control mice (Fig. 5B, C). At these doses, denatonium appears to be well-tolerated in mice, which maintained their body weight and food intake (Figure S3). Moreover, denatonium inhibited glucocorticoid-induced osteoporosis in zebrafish larvae (Fig. 5D).

## Discussion

Denatonium is a widely used bitter chemical compound (Sibert and Frude 1991). A recent study has shown that denatonium was also isolated from white kidney beans (Lee et al. 2021). Based on its relatively low toxicity and the small quantities typically used in products, denatonium has been used as aversive agents to prevent inappropriate ingestion by children and an alcohol denaturant (Berning et al. 1982; Hansen et al. 1993). Denatonium stimulated tracheal brush cells to release acetylcholine, resulting in transmitting cholinergic signaling from brush cells to sensory nerves (Hollenhorst et al. 2020, 2022). In addition, denatonium altered a variety of genes associated with acute myeloid leukemia (AML) cellular processes and inhibited the motility and migration of AML cells (Salvestrini et al. 2020). Recently, denatonium impaired the proliferation and clonogenic potential of hematopoietic stem/progenitor Cells (Pensato et al. 2024). Previously, we demonstrated that shitake mushroom (*L. edodes*) exhibits anti-osteoporotic activity by inhibiting osteoclast differentiation (Lee et al. 2020a, b). To expand on this finding, we identified a small molecule from *L. edodes*, denatonium, which leads to the inhibition of osteoclast differentiation.

Denatonium is a potent agonist of bitter taste receptors (TAS2Rs), which is broadly expressed in extra-oral tissues, the gastrointestinal tract, reproductive tissue, the brain, cancer cells, and myeloid cells (Salvestrini et al. 2020; Pulkkinen et al. 2012; Jeruzal-Swiatecka et al. 2020). A recent study highlighted the agonistic activity of denatonium toward specific TAS2R subtypes, including TAS2R105, TAS2R123, TAS2R135, TAS2R140, and TAS2R144 (Lossow et al. 2016). However, our transcriptome analysis of OCPs, regardless of whether they were treated with RANKL, revealed a notable absence of detectable expression for most *Tas2rs* (Table S3). Specifically, we observed minimal expression of *Tas2r108*, *Tas2r138*, and *Tas2r143* in OCPs. Further studies are needed to find whether the inhibitory impact of denatonium on osteoclast differentiation is dependent or independent of TAS2R signalling. Interestingly, immunostaining experiments demonstrated that denatonium is present in both the cytosol and the nucleus (Figure S4). This observation raises the possibility that denatonium might interact with intracellular proteins, alongside its known interactions with cell membrane-bound bitter receptors, though further studies are needed to confirm this hypothesis.

Phosphorylation of p65 is crucial for its nuclear translocation and transcriptional activation, with different phosphorylation sites having distinct roles: phosphorylation at p65’s serine 536 is essential for nuclear translocation, serine 529 influences transcriptional activity, and serine 276 is crucial for co-activator recruitment at target gene promoters (Mattioli et al. 2004; Wang and Baldwin 1998; Zhong et al. 1998). PI3K-AKT signalling pathway induces p65 phosphorylation via IKK activation (Yang et al. 2023; Tang et al. 2022; Bai et al. 2009; Sakurai et al. 2003; Kwon et al. 2016). Notably, denatonium inhibited AKT the phosphorylation of AKT and p65 (serine 536). This finding suggests a potential mechanism through which denatonium may exert its inhibitory effects on osteoclast differentiation. However, the exact pathway through which denatonium inhibits AKT and p65 phosphorylation remains to be elucidated.

Osteoclast differentiation is characterized by an increase in mitochondrial abundance and an elevated rate of oxygen consumption. Genes associated with the TCA cycle and mitochondrial biogenesis are upregulated during this process (Da et al. 2021; Park-Min 2018). Interestingly, denatonium inhibited the expression of genes involved in the TCA cycle and mitochondria biogenesis (Fig. 2D). A recent study revealed that NF-κB regulates key enzymes of the TCA cycle, including IDH1, IDH3A, ACO2, and SUCLA2 (Zhou et al. 2017). Our findings specifically showed that denatonium modulates the expression of the *Atp5g3* and *Idh3a* genes (Fig. 2E). This suggests a plausible mechanism in which denatonium may interfere with the localization of p65 in the promoter region of these specific genes.

Our data support a working model that highlights the regulatory role of denatonium in the process of RANKL-induced osteoclast differentiation. Following activation of the RANKL–RANK signaling pathway, the MAPK and AKT pathways activate NF-κB signaling. Consequently, p65 translocates to the nucleus, where it binds to specific gene promoters. In contrast, the presence of denatonium decreased the phosphorylation of AKT and p65, subsequently inhibiting p65 nuclear translocation and promoter enrichment at target genes. These findings support the potential application of denatonium for the treatment of osteoporosis through modulation of the pathway involved in RANKL-induced osteoclast differentiation.

## Electronic supplementary material

Below is the link to the electronic supplementary material.

Supplementary Material 1

Supplementary Material 2

Supplementary Material 3

Supplementary Material 4

## Data Availability

No datasets were generated or analysed during the current study.
