# Supplementary material for: Denatonium inhibits RANKL-induced osteoclast differentiation and rescues the osteoporotic phenotype by blocking p65 signaling pathway
Source: Mol Med. 2024 Dec 19;30:248. doi: 10.1186/s10020-024-01031-2 (PMC11660935; doi:10.1186/s10020-024-01031-2)
Supplement: Supplementary file 1 — Supplementary Material 1 [file 10020_2024_1031_MOESM1_ESM.docx]

**Supporting Information**

**Denatonium inhibits RANKL-induced osteoclast differentiation and rescues the osteoporotic phenotype by blocking p65 signaling pathway**

Sheunghun Lee, Hyerim Lee, You-Jee Jang, Kyubin Lee, Hye-Jung Kim, Jung Yeol Lee, Jin-Man Kim, Sunyou Park, Jin Sook Song, Ji Hoon Lee, Tae Kyung Hyun, Jae-Il Park, Sun-Ju Yi, and Kyunghwan Kim

**Supporting Methods**

**Osteoblast differentiation, ALP staining, and Alizarin Red S staining**

The process of osteoblast differentiation was performed as previously described (1). In brief, primary osteoblasts were isolated from newborn pups. Osteoblast precursor cells in 12-well plates were treated with ascorbic acid (50µg/mL) and β-glycerophosphate (β-GP) (10 mM), with or without denatonium treatment. On day 10, cells were fixed, and alkaline phosphatase (ALP) staining was performed by TRACP & ALP double-stain kit (Takara, USA, MK300). On day 21, alizarin red S (ARS) staining was performed with 2% ARS (pH 4.2), after ﬁxation in 4% paraformaldehyde.

**Real-time quantitative PCR**

Osteoblast precursor cells were seeded in 6-well plates and treated with ascorbic acid (50 µg/mL) and β-glycerophosphate (10 mM), with or without denatonium. On day 6, total RNA was extracted and reverse-transcribed into cDNA. Real-time PCR was performed using the iQ SYBR Green Supermix and an iQ5 real-time thermal cycler (Bio-Rad, Hercules, CA, USA). *Gapdh* mRNA levels were used as a normalization control for target gene mRNA expression. Primer sequences used for qPCR are provided in Table S1.

**Preparation of alkyne-tethered denatonium**

All reactions were monitored by thin-layer chromatography (TLC), performed using 0.2 mm silica gel plates (Merck 60 F254) and visualized by UV light (254 nm) and stain solutions, p-anisaldehyde with heating. All chemical reagents were purchased from Sigma-Aldrich, TCI or Alfa Aesar. Medium pressure liquid chromatography (MPLC) was carried out on a CombiFlash Rf system. The purity of the target compounds was determined to be >95% by analytical high-performance liquid chromatography using dual different wavelength UV detector (254 nm). Mass spectra were measured in positive electrospray ionization (ESI) mode in LCMS-2000 system (Shimadzu, Tokyo, Japan). NMR spectra were recorded on a Bruker spectrometer at 400 MHz for 1H and 100 MHz for 13C. Chemical shifts (δ) were reported in parts per million (ppm) relative to tetramethylsilane as an internal standard, and coupling constants were expressed in hertz. Final denatonium alkyne was prepared from commercially available starting material via five linear steps according to synthetic scheme.

**Immunostaining**

To examine the localization of denatonium, immunofluorescence analysis was performed. Osteoclast precursor (OCP) cells were seeded in 12-well plates at a density of 1.5 × 10^5^ cells per well. Co-treatment of denatonium alkyne (50 μM) with RANKL was carried out for 3 days. Post-treatment, cells were fixed with 4% paraformaldehyde at room temperature for 15 minutes. Subsequent permeabilization occurred at 4 °C using 1% saponin, followed by a 1-hour blocking step with 2% BSA. The cells were then incubated with TAMRA-PEG3 Azide (2) at 4 °C overnight. Following PBS washes, cells were exposed to an Alexa Fluor-conjugated secondary antibody, with incubation at room temperature for 3 hours in the dark. After triple PBS washes, cells were mounted on slides, nuclei were stained with 4′, 6-diamidino-2-phenylindole (DAPI) within ProLong Gold Antifade reagent (Invitrogen) (3). Digital images were captured using a TCS SP5 AOBS laser-scanning confocal microscope (Leica Microsystems, Heidelberg, Germany).


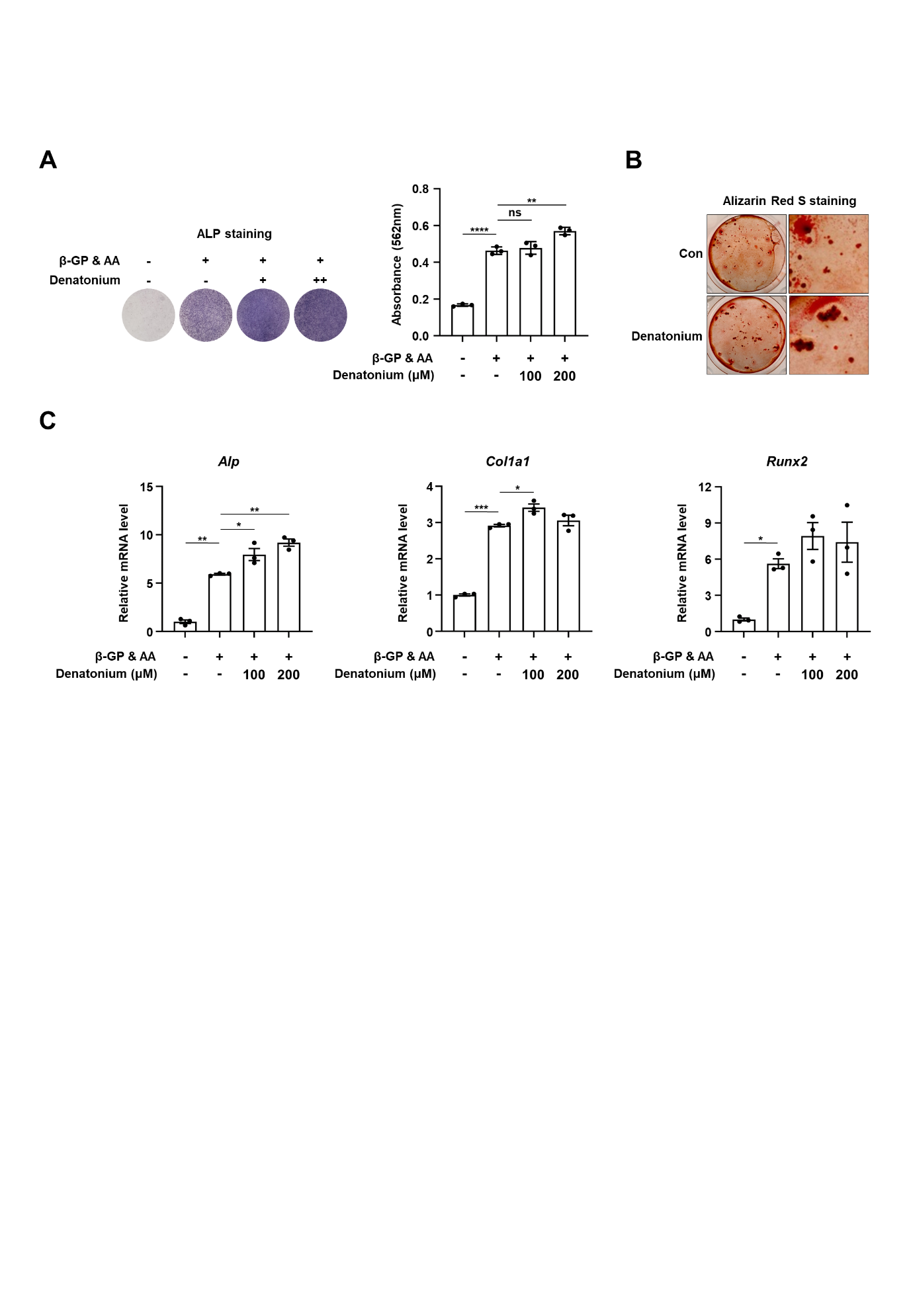


**Figure S1. Exploring denatonium’s impact on osteoblast differentiation**

**A and B** Denatonium’s effect on osteoblast differentiation: alkaline phosphatase and alizarin red S staining. Two-way ANOVA analysis, Mean±SD. *P < 0.05; ***P < 0.001. **C** Effect of denatonium on the mRNA expression of osteoblast-related genes. Relative mRNA levels of representative genes were quantified by qRT-PCR. One-way ANOVA analysis, Mean±SEM. *P < 0.05; **P < 0.01; ***P < 0.001.

**
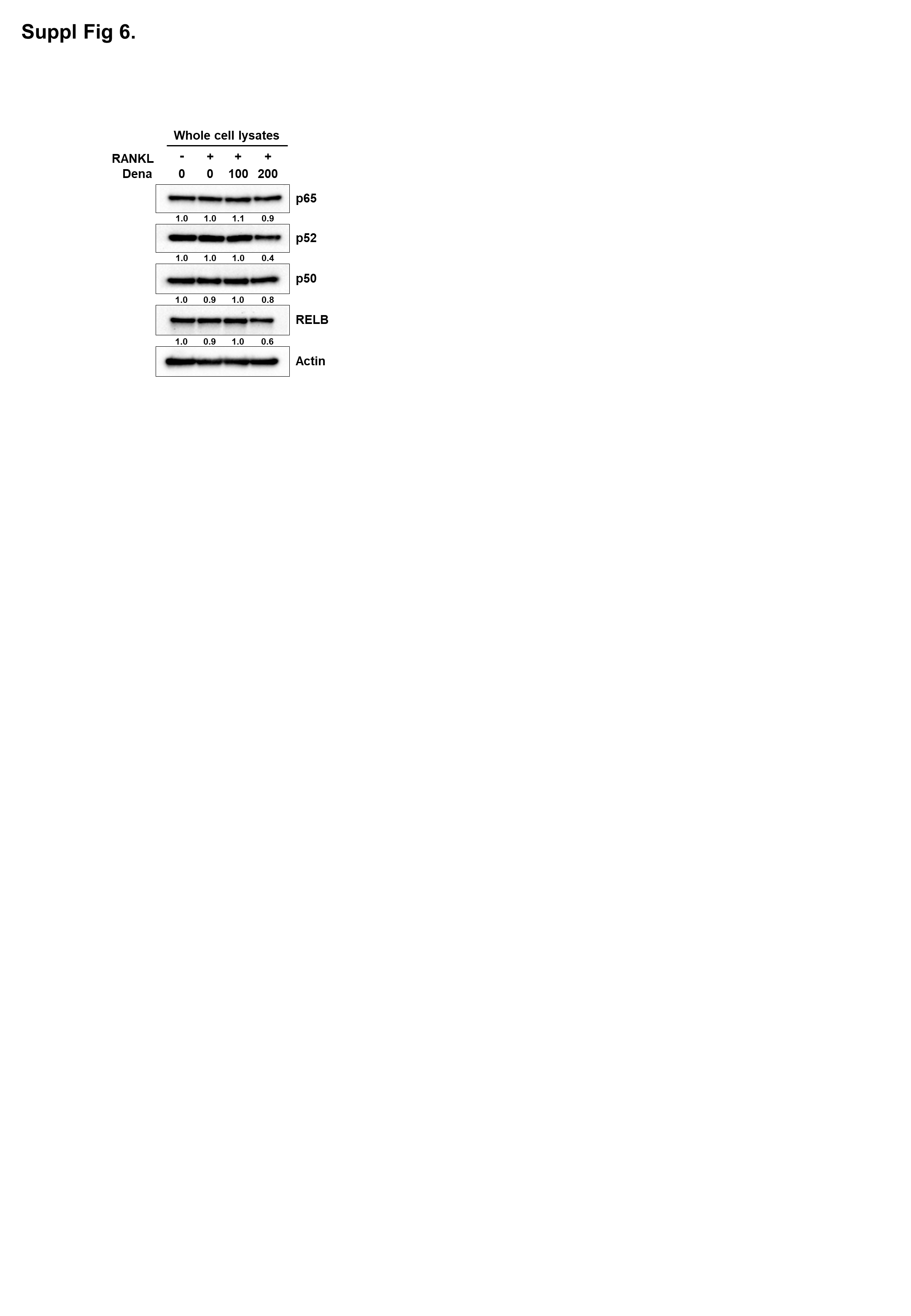
**

**Figure S2. Effect of denatonium on NF-κB expression levels**

OCPs pre-treated with denatonium were incubated with RANKL for 30 min. Western blotting was performed on whole cell lysates using the indicated antibodies.

**
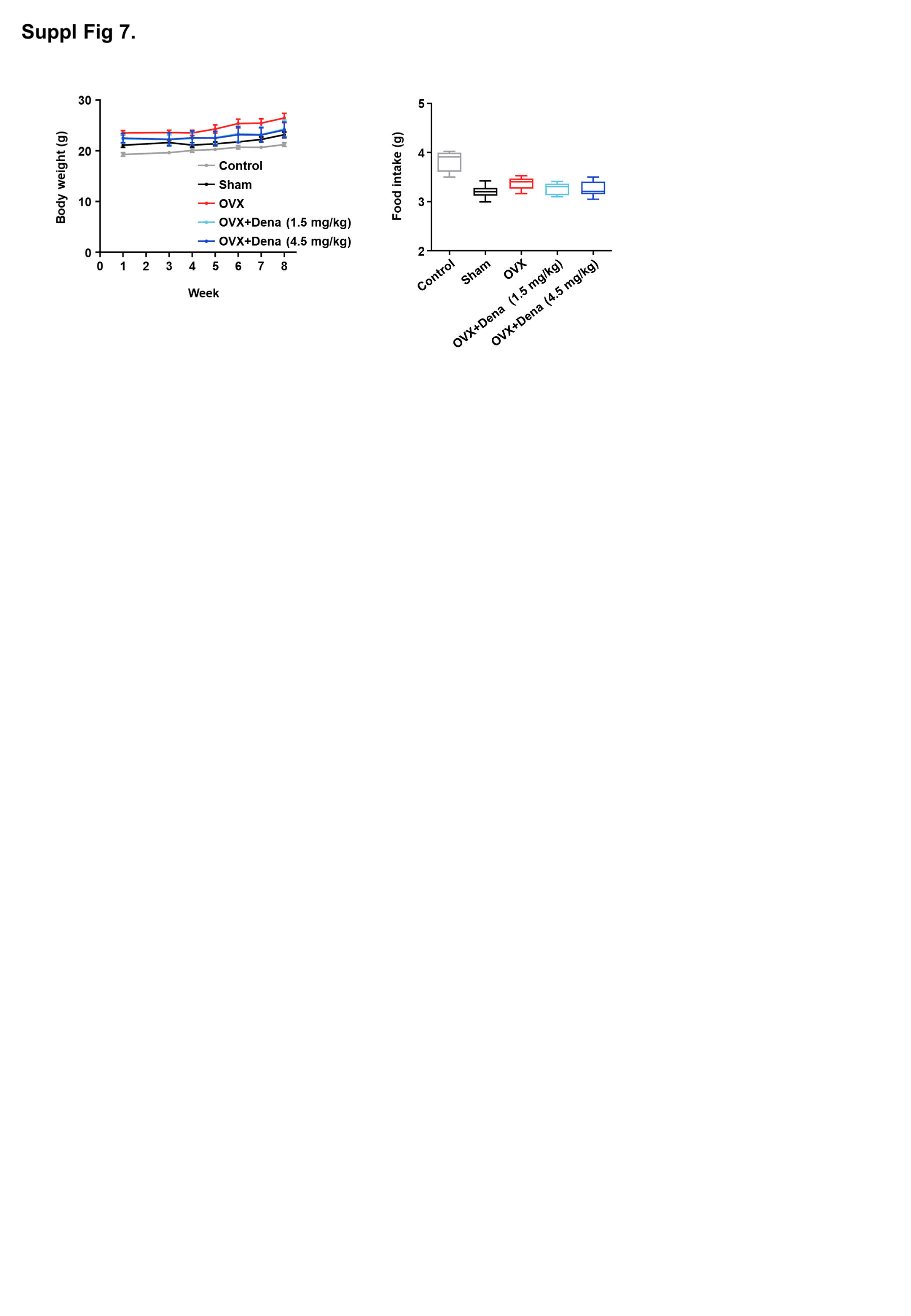
**

**Figure S3. Effect of denatonium treatment on mouse body weight and food intake**

**
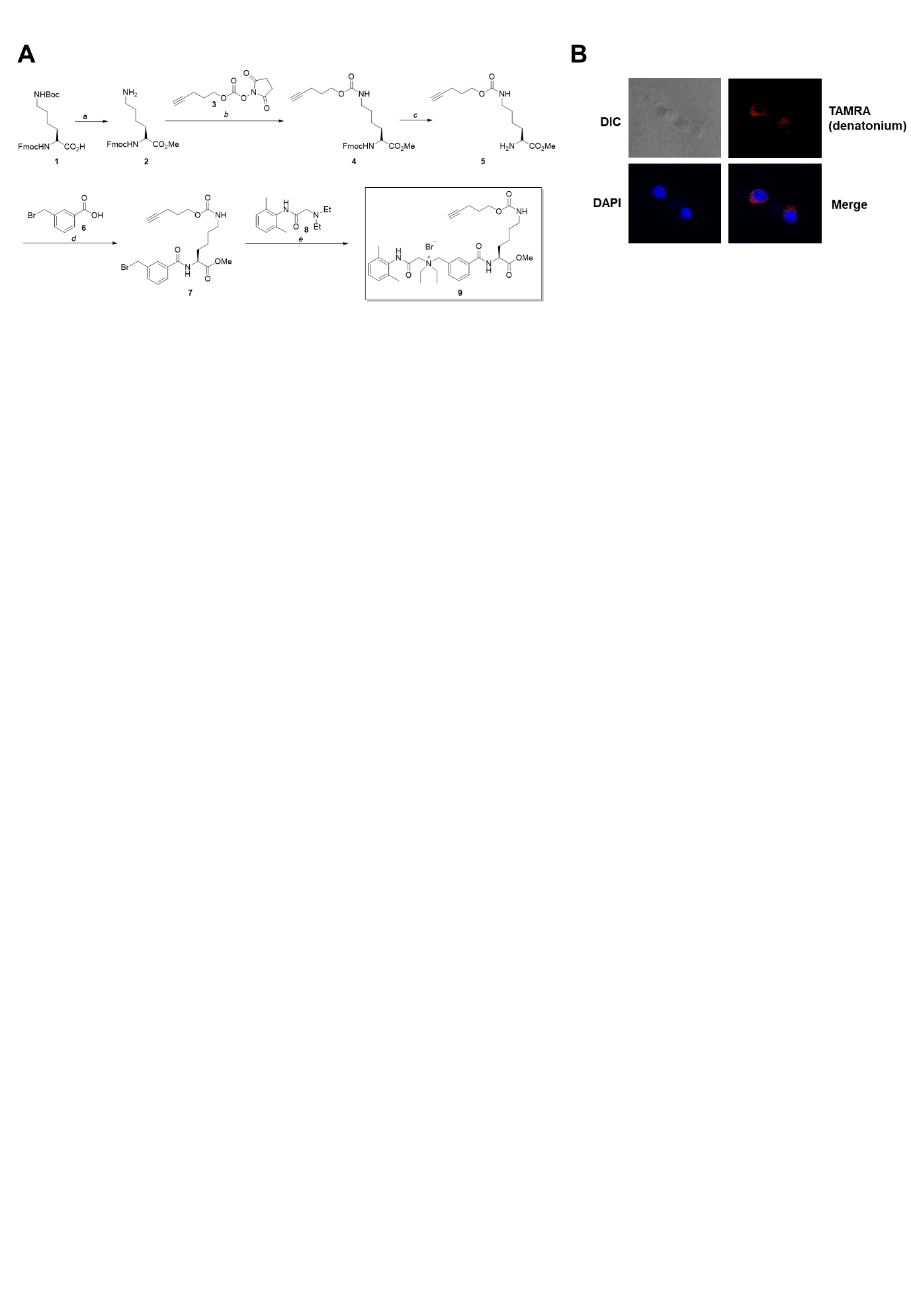
**

**Figure S4. Subcellular localization of denatonium**

**A** Synthetic scheme of denatonium alkyne. a) Thionyl chloride, MeOH, 0 ^o^C to 60 ^o^C, 2 h; TFA, rt, 10 min, 99%; b) **3**, sat’d NaHCO_3_, 0 ^o^C to rt, overnight, 83%; c) Diethylamine, MeOH, rt, 3 h, 51%; d) **6**, DCC, HOBt, DIPEA, CH_2_Cl_2_, rt, 18 h, 39%; e) **8**, MeCN, 70 ^o^C, overnight, 91%. **B** Subcellular localization of denatonium. OCP cells were seeded in 12-well plates and treated with denatonium alkyne (50 μM) with RANKL (100ng/ml) for 3 days. After treatment, the cells were fixed using 4% paraformaldehyde and subsequently incubated with TAMRA-PEG3 Azide.

**References**

1. Kim Y*, et al.* (2019) Tetracycline Analogs Inhibit Osteoclast Differentiation by Suppressing MMP-9-Mediated Histone H3 Cleavage. *Int J Mol Sci* **20**.

2. Hong V, Steinmetz NF, Manchester M, Finn MG. (2010) Labeling live cells by copper-catalyzed alkyne--azide click chemistry. *Bioconjug Chem* **21:** 1912-1916.

3. Ding M, Cho E, Chen Z, Park SW, Lee TH. (2023) (S)-2-(Cyclobutylamino)-N-(3-(3,4-dihydroisoquinolin-2(1H)-yl)-2-hydroxypropyl)isonicotinamide Attenuates RANKL-Induced Osteoclast Differentiation by Inhibiting NF-kappaB Nuclear Translocation. *Int J Mol Sci* **24**.
