# Supplementary material for: Denatonium inhibits RANKL-induced osteoclast differentiation and rescues the osteoporotic phenotype by blocking p65 signaling pathway
Source: Mol Med. 2024 Dec 19;30:248. doi: 10.1186/s10020-024-01031-2 (PMC11660935; doi:10.1186/s10020-024-01031-2)
Supplement: Supplementary file 2 — Supplementary Material 2 [file 10020_2024_1031_MOESM2_ESM.docx]

**Supplementary Table 1. Primers**

| **TARGET** | **Forward sequences (5’ → 3’)** | **Reverse sequences (5’ → 3’)** |
| --- | --- | --- |
| **RT-PCR** | | |
| *β-Actin* | GCAAGTGCTTCTAGGCGGAC | AAGAAAGGGTGTAAAACGCAGC |
| *Nfatc1* | CTCGAAAGACAGCACTGGAGCAT | CGGCTGCCTTCCGTCTCATAG |
| *Dcstamp* | CCGCTGTGGACTATCTGCTG | CTCAATGGCTGCTTTGATCG |
| *Mmp9* | CGTCGTGATCCCCACTTACT | AACACACAGGGTTTGCCTTC |
| *Atp5g3* | CCCAGAATGGTGTGTGTCAG | GCTTCAGACAAGGCAAATCC |
| *Idh3a* | GAGGTTTTGCTGGTGGTGTT | TCCTCCTGGTCCTTGAATTG |
| *Alp* | CACGGCCATCCTATATGGTAA | GAGACATTTTCCCGTTCACC |
| *Col1a1* | GACGTCCTGGTGAAGTTGGT | CAGCACCAGGAGATCCTTTC |
| *Runx2* | CCACCACTCACTACCACACG | ACTCTGGCTTTGGGAAGAGC |
| *Gapdh* | CTCCACTCACGGCAAATTCA | GCCTCACCCCATTTGATGTT |
| **ChIP** | | |
| *Dcstamp (-0.6kb)* | TTTCAAATGCTATCCCCAAA | GGCTTAGTCGGCCATCATT |
| *Mmp9 (-0.5kb)* | TCTTTCCTTCCCCAAGGAGT | CCATCCCCACACTGTAGGTT |
| *Nfatc1 (-0.7kb)* | CTCGTACAGCAAGCAATCCA | ATGTAAAATCGCAGGCTTCC |
